# Notes of the Week

**Published:** 1921-08-27

**Authors:** 


### NOTES OF THE WEEK.

### FREE-WILL OFFERINGS OR PATIENTS' PAYMENTS?

One of the questions now agitating the hospital world is the relation of patients' payments to weekly contributions. The extent to which each system is severally adopted and the possibility of combining the two are now being investigated. But it is not easy to get at the figures because two systems of patients' payments exist under two different names. In the one a fixed charge is made, but enforced only at discretion; in the other a free-will offering is asked for, but, equally at discretion, waived. Now, is there any essential difference except in name? Some hospitals like to boast that they do not adopt patients' payments. But, financially, is their revenue less from the patients than that of those hospitals which seem more exacting? It would be well to know. From the report of one hospital, which prefers to collect free-will offerings and not to impose a charge, we see that some £3,000 odd was so collected; and that half the civil patients in the hospital contributed nothing. Had patients' payments been enforced, would the amount raised have been larger? and, further, would any larger proportion of patients have contributed? If the answer to both questions is Yes, then there is a real difference and not a difference in name only. If the answer is No, may not the free-will offering be preferable?

### POLITICS AND THE IRISH HOSPITALS.

Dr. James Ashe, of Merrion Square, Dublin, has been in correspondence with Sir Hamar Greenwood concerning the position of the Irish hospitals. A few, it will be recalled, already receive a little financial assistance from the British Exchequer, but no Irish hospitals were considered by the Cave Committee, and therefore will not receive anything from the Government grant. Under the Government of Ireland Act hospitals and charities will be placed under the purview of the Governments of Southern and Northern Ireland. But Dr. Ashe does not regard that prospect without anxiety, because, he thinks, politics will take precedence of other matters. In a letter to the *Irish Independent*, he states that "moderate opinion must, sooner or later, consider the advisability of an Opposition to take in hand such problems." The future is so uncertain that comment would be idle, if not mischievous; but the proposal of an Opposition to safeguard hospital interests is remarkable and worth pondering.

### THE RADCLIFFE INFIRMARY'S EXAMPLE.

It is gratifying to learn that the half-year's finance of the Radcliffe Infirmary, Oxford, has been the best for many years. This is a tribute to the courage and initiative of the Rev. G. B. Cronshaw, the honorary treasurer, under whom the hospital's policy has become an oft-quoted example of late. Contrary to the expectation of some, the weekly contribution scheme has not interfered with the steady flow of bequests, which, if anything, are increasing. The scheme, according to Dr. Collier, has perhaps one disadvantage in that some people wish to enter the hospital directly, whereas the hospital committee is anxious that every patient should see his family doctor first. Consequently, a certain leaflet has been withdrawn by the appeal committee to remove any cause for misunderstanding. The hospital is not yet paying its way, and to reduce the overdraft some stock has been sold. In other respects good progress also has been made. A house and grounds at Cowley have been given, and will be used as a convalescent home. The Ministry of Health is making a more reasonable payment; provisions are costing a little less; the maternity home is already full; and the antenatal clinics have been a most important development. On the whole, the Radcliffe Infirmary has unquestionably strengthened its position, and is an example of the value of initiative and ideas at the present time. It is expected that £16,000 will be raised by the contribution scheme this year.

### THE ENDOWMENT OF MENTAL RESEARCH.

Mr. H. W. Burleigh, secretary of the Hospital for Epilepsy and Paralysis, Maida Vale, has taken the occasion offered by the momentary public interest in asylums to explain to the readers of the *Daily Telegraph* the frequent relation of physical disorder to mental disease. He urges the need for a "centre for the early investigation of incipient insanity," and for a well-equipped laboratory and staff. His hospital, he adds, possesses "the material and the *personnel*," but lacks the necessary funds for research, since general funds cannot be so employed. He concludes, however, "A sum of £25,000 would guarantee the expenses of a ten years' research." A timely and judicious letter, which, we hope, will be fitly rewarded.

### DOCTORS' FEES AND PATIENTS' PAYMENTS.

Following on the resolutions passed at the meeting of the British Medical Association in Newcastle, which sought to insist on a percentage of all patients' payment (other than those of paying patients) being passed to a fund at the disposal of the honorary medical staff, a letter signed by Dr. Alfred Cox, medical secretary of the British Medical Association, has been addressed to secretaries and senior honorary medical officers of all voluntary hospitals in England, Scotland, and Wales. The letter states that the British Medical Association feels that great changes are taking place in hospital finance and management, and it is essential that the honorary staffs of voluntary hospitals should establish firmly, and once for all, the principle which these resolutions enunciate; that what the honorary medical staff does with the staff fund is a matter entirely in its own discretion, but the Association's object is that the boards of all voluntary hospitals should insist on the position of their staffs being recognised in any arrangements which are made for dealing with patients who cannot properly be regarded as the recipients of charity, and should see that some payment is made, if only an acknowledgment, into the staff fund. The next step is with the hospitals, who it is to be presumed will consider this matter jointly through the British Hospitals Association and its Regional Committees. We dealt with the position raised by these resolutions in a leading article in our issue of August 6.

### THE DOCKYARD FIX AT PORTSMOUTH.

Dr. C. P. Childe and Sir Harold Pink have addressed the Portsmouth and District Trades and Labour Council on behalf of the Royal Portsmouth Hospital. Dr. Childe, criticising the system of imposing a maintenance charge upon patients, said that the best system was for those who might need the hospital voluntarily to insure themselves by small weekly contributions. If the hospital could receive 1d. per week from every class of person who used it, the committee would be able to do away with the maintenance charge. Collectors were unsatisfactory. The practical and sensible way was to agree to weekly deductions from wages. Sir Harold Pink, who followed, said that if the dockyard had come into the scheme the hospital could have carried on without debt. Representation had been provided for the dockyard, but it had not accepted it. Mr. C. Brittan contended that it was the red-tape of the Admiralty which was to blame for the abstention of the dockyard workers. At the end the Labour Council decided that its executive should draft a scheme to be laid before the next meeting. One point at all events is clear; the financial problem at Portsmouth rests with the dockyard. When that is recognised, surely so important a body will have the public spirit to join the scheme. Difficulties there may be, but the will to overcome them is the real solution of them.

### SERIOUS POSITION OF THE C.O.S.

Next month will see the last issue of the *Charity Organisation Review*, which has been carried on for thirty-six years (or forty-nine including its predecessor the *Charity Organisation Reporter*), and in its present form is a useful and informative review of various aspects of social work. As such its decease will be regretted and the regret is increased by the cause which has necessitated the end, which is explained in the August issue. The publication has always been carried on at an annual loss; that loss is increasing, and in the present critical condition of the Charity Organisation Society's finances it is no longer possible to continue expenditure which is not strictly essential. So serious is the financial position that the Society has to choose between immediate and drastic economies and the prospect of early dissolution. Some economies have already been made, and further ones can only take the form of reductions in district subsidies. The Society needs new subscribers and what is more important still more voluntary workers. It finds that the number of those who can afford to take unpaid work has greatly diminished, but not all incomes have fallen to the poverty line and not all people can or will take paid work. These voluntary workers must be discoverable somewhere, and it is to their discovery and conversion that the Society will work. We wish the Society success in its quest of workers; we do not believe that the call of personal service, if only it be rightly made, is less appealing in the present than it has been in the past.

### STANDARD COD-LIVER OIL.

Some time ago representations were made by the Glasgow Corporation to the Scottish Board of Health to take steps to have a standard for cod-liver oil in mixtures or emulsion set up. The Corporation has now been notified that the question of the inclusion in the British Pharmacopœia of an emulsion of cod-liver oil and of an extract of malt and cod-liver oil will be considered by the General Council of Medical Education and Registration during the preparation of a new Pharmacopœia, or earlier, should an addendum to the Pharmacopœia be necessary.

### THE FINANCE OF HOSPITAL MAGAZINES.

We note the continued effort to establish hospital magazines by the non-teaching hospitals, and that even those with medical schools are seeking to make their journals something more than the organ of the medical schools. The fact is interesting in many ways. Apart from its general bearing upon hospital activity and the attempt which it shows to interest a local public in the hospital's concerns, this belief in the value of publicity is commercially remarkable. The costs of newspaper production are still extremely high; the chances of making a profit from such magazines is limited; and those hospitals which note the tendency but have not themselves yielded to it are inclined to wonder whence the funds are derived to start their journals and what is their financial position after, say, their first year's experience. We do not pretend to be in the secrets of the different editorial offices, but, as we believe the movement a good one if the money can be found, we would welcome, in confidence, a business statement from those hospitals which have started journals. If they will supply us with the general facts, we will, without mention of their names, draw the moral from their figures. The information concerning the source of capital spent and the profit or loss incurred would be of general interest and encourage others to adopt a plan which on general grounds has much to recommend it. Friendly observers wish to know if a special fund is formed for the magazine, and if its accounts are kept separate or embodied under some item in the hospital's general accounts.

### A DAIRYMAN'S CONSCIENCE.

A Brighton dairyman has been summoned for having deceitful weights in his possession. The inspector visited him in July, whereupon the man calculated in decimals the sum which he had made since September last till the date of the inspector's visit. Having drawn a cheque for this sum (£3 10s. 3d.), which he sent to the Royal Sussex County Hospital, the man told the magistrates that his cheque had put him straight with his conscience. The magistrates, who imposed a nominal fine of 20s., seemed to agree, and doubtless the hospital will accept their verdict.

